# Glandular Odontogenic Cyst with Metaplastic Cartilage: Report of an Unusual Case and Literature Review

**DOI:** 10.1007/s12105-020-01239-8

**Published:** 2020-10-26

**Authors:** Hannah Crane, Bhavesh Karbhari, David Hughes, Robert Orr, Daniel Brierley

**Affiliations:** 1 Academic Unit of Oral and Maxillofacial Medicine and Pathology, School of Clinical Dentistry, 19 Claremont Crescent, Sheffield, UK; 2 Department of Oral and Maxillofacial Surgery, Chesterfield Royal Hospital, Chesterfield, UK; 3 Department of Histopathology, Sheffield Teaching Hospitals NHS Foundation Trust, Sheffield, UK

**Keywords:** Glandular odontogenic cyst, Odontogenic cysts, Diagnosis, Pathology

## Abstract

Glandular odontogenic cysts are rare odontogenic cysts with a wide range of histopathological features. In this paper we describe the clinical and pathological features of an unusual case of a glandular odontogenic cyst with metaplastic cartilage. The previous literature of odontogenic cysts presenting with metaplastic cartilage is reviewed alongside a discussion of the differential diagnoses. To our knowledge this is the first reported case of a glandular odontogenic cyst with metaplastic cartilage.

## Introduction

Glandular odontogenic cyst was first described by Padayachee and van Wyk in 1987 as a “sialo-odontogenic cyst”[1]. Gardner et al. [2] re-named the cyst as a glandular odontogenic cyst (GOC) due to the lack of evidence of a salivary origin and this nomenclature was subsequently accepted by the World Health Organisation [3]. GOC is a rare odontogenic cyst, with a demographic study showing they only account for 0.2% of odontogenic cysts within a UK population [4]. It occurs over a wide age range, with most cases diagnosed in the 5–7th decade with no gender predilection [5]. It commonly presents as a unilocular or multilocular radiolucency and is more frequently seen in the mandible [5, 6], with some studies showing a higher prevalence in the anterior regions of the jaws [5]. There are a wide range of histopathological features. Fowler et al. described 10 microscopic findings that could aid in diagnosis of GOC [5]. They suggested, following statistical analysis, that the diagnosis can be confidently made when 7 out of the 10 following features are present; eosinophilic “hobnail” cells, apocrine metaplasia, intra-epithelial microcysts, variable thickness of the epithelial lining, clear cells in the basal layer, papillary projections, cilia, multiple cystic compartments, epithelial plaque like thickenings and mucous cells [3, 5]. Magnusson et al. stated that only 0.012% of the cysts in the oral cavity fulfilled the GOC criteria microscopically [7]. GOC is important to recognise due to its high recurrence rate of 30–50% [5, 6]. Most patients are treated by invasive measures such as enucleation with or without curettage and peripheral osteotomy [8], however more aggressive treatment has been recommended for larger lesions to reduce the risk of recurrence [8].

Rarely metaplastic cartilage can be found in the cyst wall of odontogenic cysts. Previous reports of cartilage in association with both odontogenic keratocysts and orthokeratinized odontogenic cysts have been described [9–15]. In this article, we present our management of a case we believe to be the first case of a GOC with metaplastic cartilage. This is followed by a review of the literature and discussion of the differential diagnoses.

## Case report

An 89-year-old female patient was referred by her General Medical Practitioner to the Oral and Maxillofacial Surgery department. The patients presenting complaint was that of a swelling and tenderness over her right chin with a firm lump intra-orally of two to three weeks’ duration. She had been treated with a week’s course of the antibiotic Doxycycline to no benefit.

The patient’s medical history consisted of significant cardiovascular disease and advanced Dementia. Medications included psychotropic medication and the antiplatelet drug Clopidogrel. She was a lifelong non-smoker and non-drinker.

**Fig. 1:**
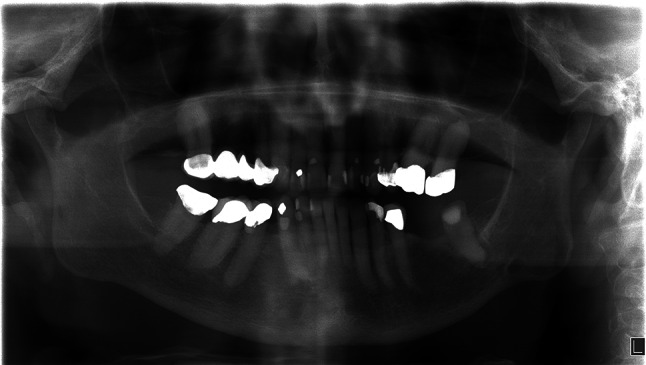
OPT radiograph showing radiolucency in the right mandible, in the region of the LL1 to LR3

Examination revealed a moderate sized firm swelling on the right chin with a corresponding intraoral component that felt fixed to bone. An orthopantomogram (OPT) showed an ill-defined radiolucency in the region of the LL1 to LR3 (Fig. 1). It was not possible to comment on the size or locularity of the lesion from the X-ray and the patients advanced dementia precluded a computerised Tomography (CT) scan.

Exploration and biopsy of the lesion was scheduled under local anaesthesia. At the time of surgery, a pale cystic lining adherent to overlying mucosa was observed with brown fluid extruding from the cavity. As it was possible to access and remove the cyst wall with relative ease, a decision was made to enucleate the cyst at this point. Curettage was performed and the cyst lining and fluid sent for histopathological examination.

**Fig. 2:**
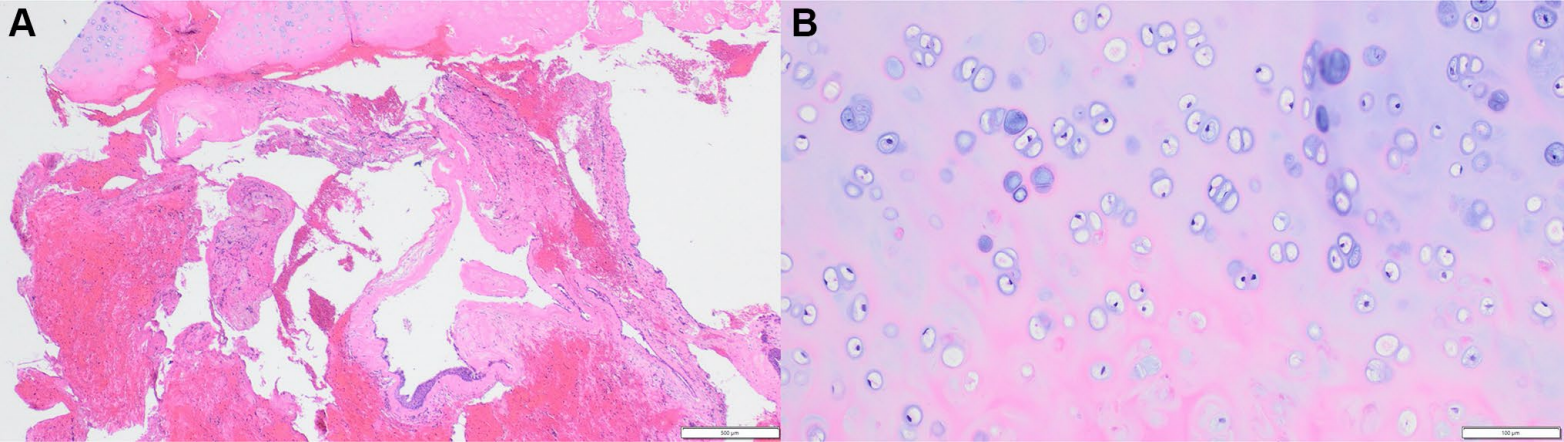
Histologically, cartilage was seen in close approximation to the cyst lining (**a**) Cyst and adjacent metaplastic cartilage, H&E stain, original magnification ×2 (**b**) Higher power view of cartilage with bland cytological features, H&E stain, original magnification ×10

**Fig. 3:**
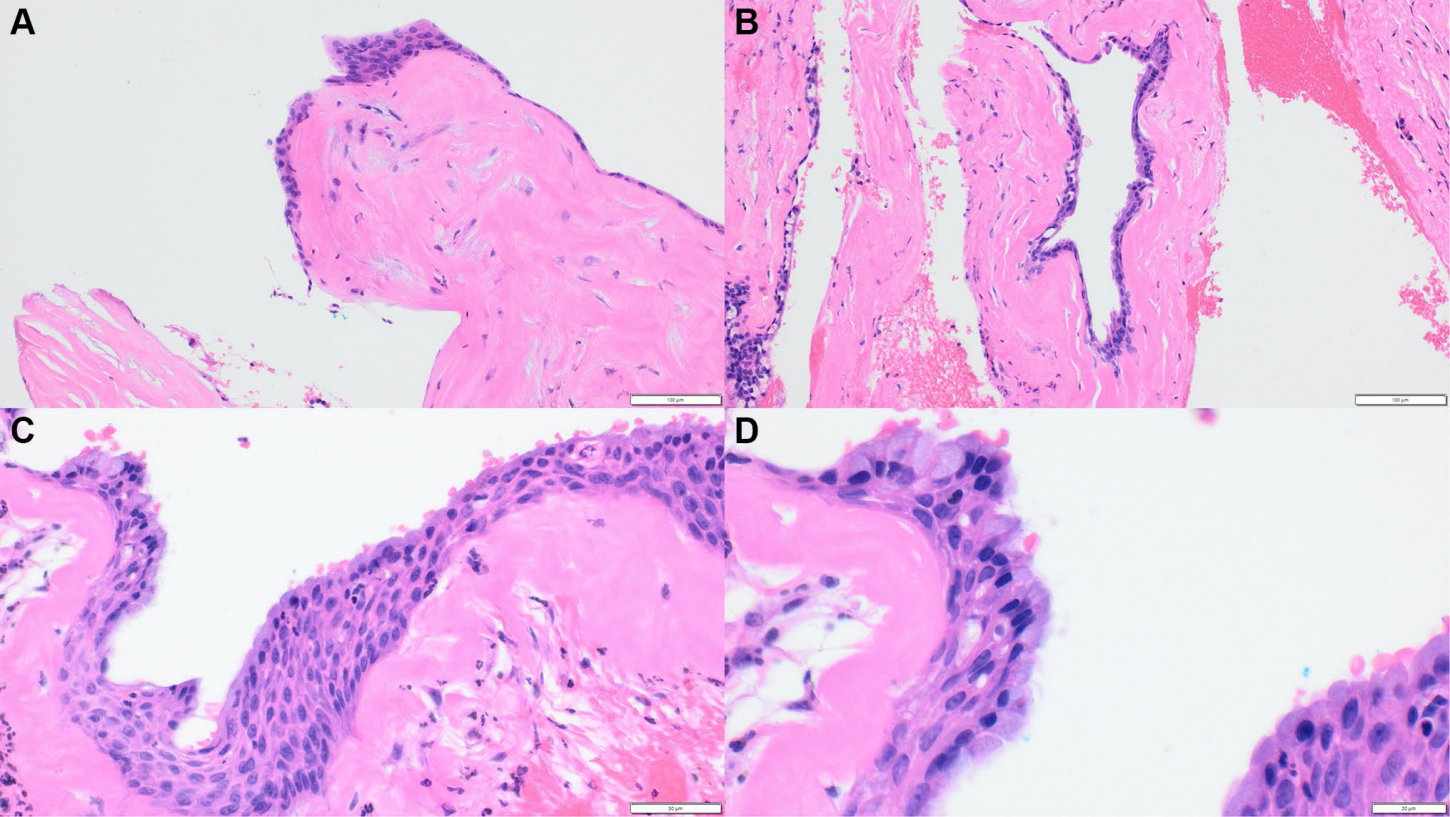
Representative pictures of the cyst lining (**a**) The cyst lining comprised stratified squamous epithelium of variable thickness, with focal plaque like thickenings, H&E stain, original magnification ×10 (**b**) Microcysts were seen in the cyst lining, H&E stain, original magnification ×10 (**c**) The cyst lining showed a superficial layer of eosinophilic hobnail cells, with mucous cells and small papillary projections also noted, H&E stain, original magnification ×20 (**d**) Higher magnification revealed mucous cells, eosinophilic hobnail cells and cilia, H&E stain, original magnification ×40

Cytopathological analysis of the cyst aspirate showed numerous neutrophils, foamy macrophages and cholesterol clefts. These features were not specific but were consistent with cyst contents. The histopathological features are seen in Figs. 2 and 3. The specimen comprised multiple fragments of fibrovascular connective tissue, which were lined by stratified squamous epithelium of variable thickness (Fig. 2a). In close association with the cystic fragments were vital lamellar bone and mature cartilage (Fig. 2). At high power, the cartilage was cytologically bland, with no evidence of cellular atypia, bi-nucleation or multinucleation, no mitotic or apoptotic activity and no evidence of infiltrative growth into soft tissue or permeative growth into bone. (Fig. 2b).

Examination of the cyst lining showed the stratified squamous epithelium to be of variable thickness with focal plaque like thickenings (Fig. 3a). The superficial layer of the epithelium was comprised of eosinophilic “hobnail” cells (Fig. 3c) and occasional microcysts were also identified (Fig. 3b). Small epithelial papillary projections were seen (Fig. 3b), with mucous cells and cilia also noted (Fig. 3d).

Given the clinically extensive swelling, presence of cartilage and to rule out higher risk pathology the lesion was reviewed by specialist musculoskeletal pathologists. They agreed the cartilage was metaplastic in appearance. A final diagnosis of a GOC with metaplastic cartilage was made.

## Discussion

GOCs are a rare entity that need fulfilment of set histopathological criteria to confirm its diagnosis. They can present as uni or multilocular, well defined or with scalloped borders, loss of cortical integrity and be associated with root resorption and unerupted teeth. When treatment planning it can be important to observe for an anterior mandibular site, multilocularity and cortical perforation as these predict the aggressive nature of GOC. Clinical and radiological (OPT and CT scans) aid in the treatment planning. Manor et al. stated only 10% of reported cases used CT scans preoperatively [16]. CT scans are now more readily available and aid in appropriate treatment of a cyst that has the propensity of at least a 30% recurrence rate [5, 6]. In particular, it characterizes size, locularity and cortical perforation—the latter occurring at a rate higher than odontogenic keratocysts [17].

Lesion size is deemed not to be a good predictor of recurrence as both recurrent and non-recurrent cysts were large in more that 75% of cases [8]. The aggressive behaviour and increased risk of recurrence may be associated with cell kinetics in the lining epithelium such as microcysts, infolding and plaques which suggest active cell proliferation [6]. It is therefore important to elucidate this by incisional biopsy prior to definitive treatment of potential GOC’s. This of course must be balanced against the risks of general anaesthesia required in aggressive subsequent treatment in those with medical co-morbidities and advanced age as in the presented case. It is important to undertake a tissue biopsy rather than an aspirate alone as suggested by one study [18]. In our case and that of another [19] an aspirate did not reveal any specificity towards GOC.

Various treatment modalities have been advocated for GOC’s ranging from enucleation with curettage, application of Carnoy’s solution, two-stage marsupialization with second surgery, peripheral ostectomy and en bloc jaw resection. The latter treatment showing no recurrences as described previously [8]. The most commonly reported treatment modalities are enucleation, curettage and peripheral ostectomy [20, 21]. Marginal/partial jaw resection has been advocated in higher risk multilocular, large lesions with compromised jaw border integrity or proximity to vital structures. Aside from en bloc resection, the treatment still has a recurrence rate of around 29.2% within 0.5 to 7 years (mean 2.9 years) [8]. It is therefore important to review the patient clinically and radiographically for at least 3 to 7 years’ dependent on the risk stratification.

### Glandular odontogenic Cyst with a Cartilaginous Component

Odontogenic cysts with metaplastic cartilage are extremely rare, with only ten cases previously reported in the literature [9–15]. Details of these cases are seen in Table 1 and the majority of the previously reported cases showed cartilage in association with an odontogenic keratocyst (OKC) [9, 10, 12–15], with only one case reporting cartilage in association with an orthokeratinised odontogenic cyst (OOC) [11]. The previously reported cases occurred over a wide age range (16–66 years), however the majority of cases occurred in the 5-7th decades with no gender predilection [9–15]. Seven cases occurred in the mandible and where treatment details were given, most cases appeared to be treated relatively conservatively [9–15]. Six cases provided follow up detail and three of these cases reported recurrences over a 2–9 year period [9–15], however the number of reported cases is too small to conclude whether the recurrence rate differs from OKC without metaplastic cartilage. To our knowledge, the case presented here is the first case of metaplastic cartilage in the cyst wall of a glandular odontogenic cyst.

**Table 1:** Details of previous case reports of odontogenic cysts with associated cartilage [9–15].

Author	Cyst	Age	Gender	Location	Treatment	Recurrence
Fornatora et al. [9]	OKC	66	Male	Mandible	Initially treated with enucleation. Recurrence treated with en-bloc resection	Initially recurred after 2 years. No recurrence during a 16-month follow-up after en-bloc resection
Vicente-Barrero et al. [10]	OKC	44	Female	Maxilla	Unknown	Unknown
Ide et al. [11]	OOC	38	Male	Mandible	Enucleation	No recurrence following 3 years follow up
Mosqueda-Taylor et al. [12]	OKC	48	Female	Mandible	Initially treated with curettage. Recurrence treated with enucleation and excision of surrounding bone	Initially recurred after 3 years. Lost to follow up 2 months after subsequent enucleation
Yih and Krump [13]	OKC	53	Male	Maxilla	Enucleation	Unknown
Kratochvil and Brannon [14]	OKC	60	Female	Mandible	Unknown	Lost to follow up
Kratochvil and Brannon [14]	OKC	48	Male	Maxilla	Initially treated with surgical excision. Treatment of recurrence unknown	Recurrence occurred 9 years after initial surgery
Kratochvil and Brannon [14]	OKC	59	Male	Mandible	Marsupialization	No recurrence following 2 months follow up
Kratochvil and Brannon [14]	OKC	16	Female	Mandible	Unknown	Lost to follow up
Arwill and Kahnberg [15]	OKC	59	Male	Mandible	Initially treated endodontically, followed by apicectomy, incision and drainage and re-enucleation	No recurrence following 4 years follow up

Kratochvil and Brannon [14] discussed a number of possible explanations for the co-existence of cartilage in the walls of OKCs including; co-existence of a benign chondroma, persistence of vestigial remnants of Meckel’s cartilage, metaplastic change of the fibrous connective tissue in response to chronic inflammation and induction of cyst wall by the epithelial lining [14]. Benign chondromas are extremely rare in the gnathic bones [3] and therefore we feel that it is unlikely that the case presented here represents a collision of a GOC with a benign chondroma. Remnants of Meckel’s cartilage are also a possible explanation for presence of cartilage and cannot be entirely ruled out. We feel that the current case most likely represents metaplastic change due to chronic irritation, as the cartilage is not seen in direct contact with the epithelial lining, with features similar to a previous report of an OOC with cartilage within the cyst wall which was considered heterotopic [11]. However, the explanation for the presence of cartilage in the wall of odontogenic cysts is of academic interest only and the change has been customarily explained by cartilaginous metaplasia in the cyst wall [11], but other explanations cannot be entirely excluded.

When cartilage is encountered in the wall of an odontogenic cyst, there are a number of differential diagnoses that are important to consider. Due to the benign appearance of the cartilage a chondroma can be considered in the differential diagnosis, however they are extremely rare in the head and neck region and the presence of any cartilage should raise the possibility of malignancy [3]. Chondrosarcoma is also extremely rare in the head and neck, accounting for only 0.1% of all head and neck neoplasms [22]. Chondrosarcoma generally shows destructive growth, invading the surrounding bone [3]. Histologically they appear identical to chondrosarcomas occurring elsewhere in the body, comprising of lobules of blue-grey cartilaginous matrix [23], with increasing cellularity, mitoses and atypia as the grade increases [23]. A chondroblastic osteosarcoma also needs to be excluded, as these are much more common in the jawbones and have a worse prognosis in comparison to chondrosarcoma [24]. Therefore it is important to look for the presence of malignant osteoid which will lead to a diagnosis of chondroblastic osteosarcoma [24].

Nasopalatine cysts are also reported to commonly contain cartilage within their wall, however these are site specific and are only encountered in the anterior maxilla [25]. Finally, a diagnosis of a teratoma can also be considered, however a teratoma will have tissues from all three germ cell layers (ectoderm, endoderm and mesoderm), which will not be seen in an odontogenic cyst with metaplastic cartilage [26]. Another important differential diagnosis of a GOC is an intraosseous mucoepidermoid carcinoma. Intraosseous mucoepidermoid carcinoma presents with the classical mix of epidermoid, intermediate and clear cells typically in a solid and cystic pattern [3]. In the presented case there were no areas concerning for intraosseous mucoepidermoid carcinoma and epidermoid cells were not identified, whereas the classical GOC hobnail cells, cilia and plaque like thickenings were seen (Fig. 3). However, in some situations it can be challenging to differentiate between a GOC and an intraosseous mucoepidermoid carcinoma and molecular testing for a MAML2 gene rearrangement may be useful in these circumstances [27].

## Conclusions

This article presents a case of GOC and our management along with a review of the literature. In particular, this report describes an unusual case of a glandular odontogenic cyst with metaplastic cartilage which raises awareness of this uncommon occurrence.
